# Long-Term Decrease in VLA-4 Expression and Functional Impairment of Dendritic Cells during Natalizumab Therapy in Patients with Multiple Sclerosis

**DOI:** 10.1371/journal.pone.0034103

**Published:** 2012-04-04

**Authors:** Clara de Andrés, Roseta Teijeiro, Bárbara Alonso, Francisco Sánchez-Madrid, M. Luisa Martínez, Juan Guzmán de Villoria, Eduardo Fernández-Cruz, Silvia Sánchez-Ramón

**Affiliations:** 1 Department of Neurology, Hospital General Universitario Gregorio Marañón, Madrid, Spain; 2 Department of Immunology, Hospital General Universitario Gregorio Marañón, Madrid, Spain; 3 Department of Immunology, Hospital de la Princesa, Madrid, Spain; University of Lyon, France

## Abstract

Myeloid and plasmacytoid dendritic cells (mDCs, pDCs) are central to the initiation and the regulation of immune processes in multiple sclerosis (MS). Natalizumab (NTZ) is a humanized monoclonal antibody approved for the treatment of MS that acts by blocking expression of VLA-4 integrins on the surface of leukocytes. We determined the proportions of circulating DC subsets and analyzed expression of VLA-4 expression in 6 relapsing-remitting MS patients treated with NTZ for 1 year. VLA-4 expression levels on pDCs and mDCs decreased significantly during follow-up. In vitro coculture of peripheral blood mononuclear cells and pDCs, with different doses of NTZ in healthy controls (HC) and MS patients showed dose-dependent down-regulation of VLA-4 expression levels in both MS patients and HC, and reduced functional ability to stimulate antigen-specific T-lymphocyte responses. The biological impact of NTZ may in part be attributable to inhibition of transmigration of circulating DCs into the central nervous system, but also to functional impairment of interactions between T cells and DC.

## Introduction

Multiple sclerosis (MS) is a multifocal chronic inflammatory perivascular demyelinating disease of the central nervous system (CNS) that is thought to be immune-mediated. Inflammatory infiltrates in MS lesions consist mainly of macrophages, CD8 cells, CD4 cells, and B cells, all of which play a role in the disease [1]. The most accepted hypothesis is that peripheral activated self-reactive lymphocytes invade the CNS through the blood–brain barrier (BBB) and, together with the resident microglia and/or dendritic cells (DCs), elicit local CNS immune responses and cause demyelination and damage to oligodendrocytes and axons [2]. DCs are central to the maintenance of immunological tolerance, as well as the initiation and regulation of immune responses. DCs, and plasmacytoid DCs (pDCs) in particular, are found in MS lesions [3] and are functionally altered in patients with MS [4], [5]. Circulating DCs comprise at least 2 well-characterized subsets, namely myeloid DCs (mDCs) and pDCs, which are distinguished by their function and by their reactivity with a panel of monoclonal antibodies. mDCs are a major subpopulation of blood DCs that are CD4^+^Lin^−^CD11c^+^CD123^dim^CD45RO^+^HLA-DR^+^. They express myeloid markers (CD13, CD33) and have a monocytoid appearance. A major subset of mDCs also expresses the CD1c (BDCA-1) antigen and drives differentiation of T cells into Th1 lymphocytes. Blood pDCs express a specific CD303 (BDCA-2) marker. Phenotyping of peripheral BDCA-2^+^ DCs characterizes these cells as being CD4^+^Lin^−^CD11c^−^CD123^bright^CD45RA^+^ and lacking myeloid lineage markers. Both DC subsets and the inflammatory environment in which DCs become activated can influence the type of T-cell response they elicit and thus contribute to MS lesions. In addition, both subsets have been found in the cerebrospinal fluid (CSF) of MS patients, especially during relapses [6], [7], [8].

Transmigration of immune cells into the CNS is regulated by chemokines, cytokines, and adhesion molecules on immune cells. Breakdown of the BBB is well documented in the pathogenesis of MS [9]. In MS lesions, activated endothelial cells express abnormally elevated levels of adhesion molecules, including intercellular adhesion molecule I (ICAM-I) and vascular cell adhesion molecule I (VCAM-I), and their elevated levels correlate with the extent of immune cell infiltration. Ligands for these adhesions molecules, lymphocyte function-associated antigen 1 (LFA-1) and very late antigen 4 (VLA-4) integrins, respectively, have been identified on perivascular inflammatory cells in MS lesions [10], [11], [12]. VLA-4 (CD49d/CD29), the α-4 submember of the β1 integrin family expressed on most mononuclear hematopoietic cells, plays a role in several immunological tasks, including immune cell trafficking, activation of myeloid cells and naïve T and B lymphocytes, and differentiation of effector T cells into Th1, Th2, or Th17 [13]. Indeed, VLA-4 constitutes an essential part of the immunological synapse, and binding of VLA-4 provides costimulatory signals to T cells [14]. LFA-1 (CD11a) mediates cell adhesion by binding to a family of adhesion molecules, including ICAM-1, whose expression reflects activation of cells. [15]. Natalizumab (NTZ) is a humanized monoclonal antibody (mAb) that binds to the α4β1 chain subunit of VLA4 and a4-α4β7 integrins on peripheral immune cells and blocks the interaction of VLA-4 with its natural vascular ligands (VCAM-I). Both integrins (VLA-4 and LFA-1) can potentially regulate extravasation of blood cells into inflamed tissue and differentiation, priming, and proliferation of T cells. As a consequence, trafficking of leukocytes through the BBB into the CNS and, therefore, inflammatory MS processes are attenuated by NTZ [16]. Of particular interest is the effect of NTZ on DCs, as DCs are increasingly recognized as an important component in MS pathogenesis [17].

NTZ was approved for treatment of relapsing-remitting MS (RRMS) based on the results of 2 phase III clinical trials and an evidence-based review [18], [19], [20] in which both the relapse rate and magnetic resonance imaging (MRI) activity were shown to decrease. The biological effects of NTZ on different immune cell populations include decreases in the counts of CD4^+^ and CD8^+^ T lymphocytes, CD19^+^ B cells, and CD138^+^ plasma cells in CSF; NTZ is also responsible for decreases in functional surface expression of α4-integrin in these cells and in the migration capacity of circulating CD4^+^ and CD8^+^ lymphocytes [21], [22], [23]. NTZ induces the release of immature leukocytes from bone marrow and increases circulating bone marrow–derived CD34^+^ cell expression of VLA-4 [24]. However, very little is known about the effect of NTZ on DCs, which are key cells in immune homeostasis. To determine whether circulating DCs are affected by NTZ, we prospectively investigated the circulating proportion of mDCs and pDCs and their expression of VLA-4 and LFA-1 molecules in RRMS patients before starting treatment with NTZ (baseline) and after 48 hours, and 1, 3, and 12 months of treatment. We then compared these data with those of non-active and non-treated RRMS (MSC) patients and healthy controls (HC).

## Materials and Methods

### Patients

We prospectively enrolled 6 patients (4 women, 2 men) with clinically diagnosed RRMS [25] who were treated with NTZ because of at least 1 relapse during the previous year on treatment with another immunomodulatory agent (interferon-β and/or glatiramer acetate) before receiving NTZ, with a minimum of 9 T2 lesions on cranial MRI or at least the presence of almost 1 active (Gd^+^) lesion (according to the European Medicine Agency authorization). A control group was formed from patients with non-active RRMS who had never received immunomodulatory therapy (n = 4) and healthy controls HC (n = 10). Relapse was defined as the appearance of new symptoms or reappearance of 1 or more neurological symptoms that persisted for at least 48 hours and that had been preceded by at least 30 days of stable or improved neurological status in the absence of fever or infection. The annualized relapse rate (ARR) was calculated as the number of exacerbations per year/number of patients and was assessed in the year previous to NTZ treatment and during the year of treatment. Disease severity was scored using Kurtzke's Expanded Disability Status Scale (EDSS) [26]. Routine laboratory blood tests and urinalysis were performed, and adverse events and concomitant therapy were monitored. NTZ was administered intravenously at a dose of 300 mg every 4 weeks. Patients were examined and changes in the EDSS score were recorded during follow-up. At each visit, we recorded relapses, adverse events, and occurrence of infections. Peripheral blood samples were obtained at baseline, 48 hours, and at 1, 3, and 12 months after the start of NTZ therapy. The latter samples were obtained before drug administration. Patients were requested to report new symptoms or adverse events to our clinic and were revaluated. The Ethics Committee of the hospital approved the protocol, and all subjects provided their written informed consent.

At enrolment, none of the patients had experienced an acute relapse or had received corticosteroids for at least 1 month before and none had received immunosuppressive or immunomodulatory drugs during the 2 months. None of the patients were treated with immunomodulatory agents during NTZ treatment. At entry, all patients were free of signs or symptoms suggestive of immune compromise or opportunistic infection, based on their clinical history and the results of a physical examination and laboratory tests. We excluded patients with other inflammatory or autoimmune diseases, any severe systemic illness, or abnormal results in a routine laboratory investigation (blood chemistry, complete blood count, C-reactive protein, thyroid function test, anti-nuclear antibodies, HIV, hepatitis B and C antibodies, and urinalysis). A single baseline sample was collected from MSC and HC at the same time as blood was collected from NTZ-treated MS patients.

### Evaluation of MRI active images

Weighted MRI scans of the brain were obtained at baseline and after 6 and 12 months of NTZ. The number of Gd+ lesions was analyzed using dual echo fast/turbo spin echo sequences providing scans with proton density–weighted, T1-weighted, and T2-weighted contrast while the patients were on NTZ. All scans were performed at 1.5 Tesla. T2-weighted and gadolinium-enhanced T1-weighted MRI scans were not performed within 4 weeks of corticosteroid treatment. Non-active radiological disease was defined as no gadolinium-enhancing lesions and no new T2-hyperintense lesions [27].

### Blood sampling and phenotyping of peripheral blood DCs. Analysis of VLA-4 and LFA-1 expression levels during therapy with NTZ

Venous blood samples were collected in pre-chilled ethylenediamine tetra-acetic acid (EDTA) sterile tubes and processed within 2 hours of collection. A complete blood count was also performed. Whole peripheral blood samples (100 µL) were labelled by direct staining with mAbs and, after incubation, were lysed with FACS Lysing solution. Expression of α-4 (CD49d [clone HP2/1, FITC]) and β-1 (CD29, [clone 4B4, FITC]) integrin subunits were investigated on DCs from Becton Dickinson (BD Immunocytometry Systems, San José, California, USA).

The mononuclear cell analysis region was analyzed for DC markers and cell debris, and dead cells were excluded from the analysis based on scatter signals. mDCs were defined as lineage- (CD19-CD3-CD14-CD56-CD16-) and HLA-DR+BDCA-1+ cells. pDCs were defined as Lin-HLA-DR+BDCA-2+CD123+ (IL-3 receptor α-chain). Percentages of each cell subset are expressed with respect to total mononuclear cells (R1). Cell subsets and the expression levels of adhesion molecule VLA-4 and LFA-1 subunits on circulating mDCs and pDCs were acquired and analyzed using 4-colour flow cytometry. Directly conjugated mAbs and FACS Lysing solution were obtained from Becton Dickinson. Isotype control mAbs were used to determine background fluorescence levels. Acquisition and analysis were performed in a FACSort flow cytometer (Becton Dickinson) using CellQuest and Flow Jo software. Fluorescence intensity gains in cellular surface markers are a measure of surface expression levels of the investigated molecules of HC and MSC patients, as previously described [28], [29].

### Culture assays

We incubated peripheral blood mononuclear cells (PBMCs) from HC and MS patients treated with increasing amounts of NTZ. The local ethics committee approved the performance of a study involving human cells. PBMCs were obtained from HC and NTZ-treated MS patients at baseline, and in NTZ-treated MS patients at specific treatment intervals (48 hours, 3-, 6- and 12- months) by Ficoll density gradient centrifugation. To test the hypothesis that NTZ modulates DC counts and VLA-4 and LFA-1 expression *in vitro*, PBMCs were cultured in the presence or absence of NTZ at 3 different doses (22, 110, and 550 µg/mL, respectively). We chose a natalizumab concentration of 110 µg/mL because it represents the mean maximum observed serum concentration following the repeat intravenous administration of a 300 mg dose of natalizumab to MS patients according to the pharmacokinetic studies shown in the package insert (Tysabri (natalizumab) [package insert]. Cambridge, MA: Biogen Idec Inc.; July 2010.). We then used a curve of comprising one point of 1/5 of 110 (22 µg/mL) and an additional point at 5 times 110 (550 µg/mL). Moreover, 23 µg/ml is considered the mean average steady-state trough natalizumab concentrations over the dosing period.

### Antigen-specific T-lymphocyte proliferation assay

We purified pDCs using magnetic cell sorting (BDCA-2 cell isolation kit, MiltenyiBiotec) from freshly drawn whole blood of HC and NTZ-treated MS patients, obtaining more than 94% cells with a lin-1^−^CD123^high^MHC class II^high^ phenotype. pDCs were cultured in the presence of antigen-specific stimulus (purified protein derivative [PPD]) (50 IU/mL) or no antigen, and, 4 hours later, cells were treated with interleukin (IL) 3 (10 ng/mL) and NTZ at 110 µg/mL for 1 more day to obtain mature pDCs. Autologous PBMCs were labelled with carboxyfluorescein succinimidyl ester (CFSE). Pulsed mature pDCs were washed, counted, and adjusted at a DC∶T cell ratio of 1∶10 in 300-µL final volumes of medium (RPMI 1640 supplemented with autologous serum). Assays were performed in triplicate. After 6 days of coculture, lymphocyte proliferation was assessed using the CFSE dilution method.

In parallel, we performed proliferation assays in PBMCs labelled with CFSE that were stimulated or not with PPD (50 IU/mL) and assessed lymphocyte proliferation using the CFSE-dilution method at day 6.

### Statistical analysis

Descriptive data are presented as mean ± standard deviation (SD) and median and interquartile range (IQR). Comparisons between patients and controls were performed using the non-parametric Mann-Whitney test and the 2-tailed Fisher exact test, when necessary. For comparison of paired data at baseline (before NTZ treatment) and after therapy, we used an analysis of variance for repeated measures taking into account all the time points and without assuming a Gaussian distribution (Kruskal-Wallis test) and Dunn's multiple comparison test. Differences were considered statistically significant with p values<0.05 and highly significant with p<0.01 and p<0.001. All statistical analyses were performed using SPSS (SPSS, Inc, Chicago, Illinois, USA).

## Results

Patient characteristics at the initiation of treatment are presented in Table 1. The study cohort comprised 6 patients with RRMS. Mean age was 33.8 years (range, 26–50 years) and mean disease duration was 8.2 years (range, 2–17). The mean ARR was 2.4 (range, 1–4) in the previous year. The median EDSS score at study entry was 2.5 (range, 1.5–5.0). The median number of Gd-enhancing lesions prior to NTZ treatments was 4.5 (range, 0–15). The median interval between discontinuation of previous treatment and start of NTZ was 65 days (range, 35–125 days). All patients were previously on immunomodulatory treatment with interferon or glatiramer acetate before receiving NTZ, and 2 patients had also received mitoxantrone induction 3 and 4 years before the immunomodulatory treatment. With respect to the MSC group, mean age was 34.0 years (range, 18–52 years) and mean disease duration was 9.6 years (range, 3–25). The mean ARR was 1.2 (range, 1–2) in the previous year. The median EDSS score at study entry was 1.2 (range, 0–4). For the HC group, the mean age was 32.1 years (range, 29–35 years), 30 were women and 2were men.

**Table 1:** Demographic and clinical characteristics of patients with relapsing-remitting multiple sclerosis (n = 6).

Mean age, years (range)	33.8 (26–50)
Mean disease duration, years (range)	8.2 (2–17)
Mean annualized relapse rate (ARR) during the previous year, mean (range)	2.4 (1–4)
Mean EDSS (range) before treatment	2.5 (1.5–5.0)
Gender, female/male	4/2
Number of Gd-enhancing lesions before NTZ	4.5 (0–15)

Definition of relapsing-remitting multiple sclerosis according to Poser et al. [25] ARR = annualized relapse rate. EDSS = Expanded Disability Status Scale (Kurtzke).

### Clinical efficacy assessment

One patient with very active disease (clinically and on MRI) suffered an early relapse after the first NTZ dose, as described by other authors [30]. The patient was treated with intravenous methylprednisolone 1 g/day for 5 days. Clinical remission was incomplete. The 5 remaining MS patients were clinically stable at 6 and 12 months. The mean number of Gd-enhancing lesions in MRI decreased significantly from 4.5 to 0.57 at 12 months of NTZ. Our data confirm that active MRI findings in RRMS patients were clearly modified by NTZ treatment. Headache and fatigue were the most common clinical adverse reactions to NTZ. Two patients complained of hypersomnia on the day of NTZ infusion.

### In vivo effects of NTZ on DC subset proportions and VLA-4 and LFA-1 surface integrin expression during follow-up in MS patients

**Figure 1:**
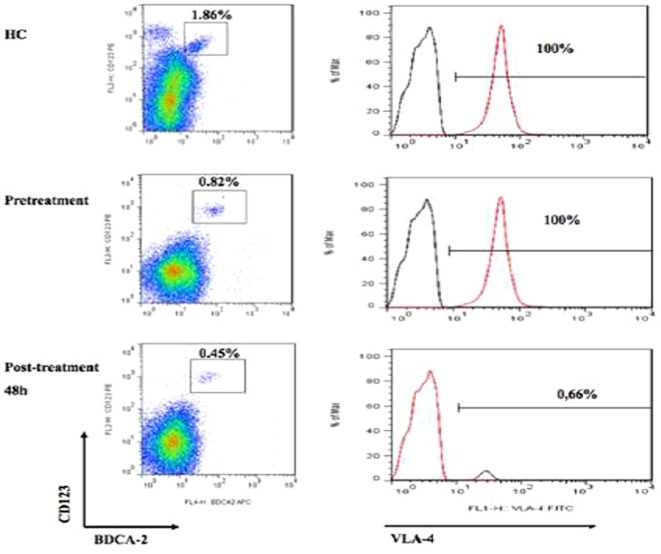
Changes in the in vivo expression of VLA-4 on pDCs before and after 48 hours of natalizumab (NTZ) therapy in MS patients, compared with healthy controls (HC). Isotypic IgG4 control is marked in red. The example shown is representative for data obtained from the 6 RRMS patients and 10 HC.

At baseline (pre-treatment), no statistically significant differences were observed in VLA-4 and LFA-1 expression on the mDCs between NTZ-treated MS patients and HC or MSC patients. Interestingly, proportions of mDCs were higher in NTZ-treated MS patients at baseline than in HC (p = 0.04) (Table 2). Similarly, LFA-1 expression on mDCs was lower in NTZ-treated MS patients than those of HC and MSC patients (Table 2). LFA-1 expression on pDCs was lower in MS patients at baseline (NTZ and MSC) than in HC (p = 0.06) (Table 2). Analysis of fluorescence of VLA-4 and LFA-1 on DC subsets during follow-up samples after short-term therapy (48 h), intermediate therapy (3 mo), and long-term therapy (12 mo) revealed a statistically significant decrease in VLA-4 in pDCs and mDCs between baseline and 48 hours (97.00±6.22 *vs* 13.41±31.35 for pDCs and 98.39±3.25 *vs.* 26.22±24.19 for mDCs; p = 0.04 both), at 3 months (13.73±30.44 for pDCs and 8.58±13.99 for mDCs; p<0.0001 both); and at 12 months (0.28±0.51 for pDCs and 14.57±19.81 for mDCs; p = 0.003 and 0.004, respectively) (Table 2). In Fig. 1, the dot-plot shows the in vivo decrease at 48 hours in VLA-4 on pDCs (from 0.82% to 0.45%) after starting NTZ treatment in a representative patient. No changes in LFA-1 were noted on pDCs, whereas LFA-1 significantly increased on mDCs at 12 months (17.55±16.41 *vs.* 47.47±36.71; p = 0.05) (Table 2). There was a trend toward a decreasing proportion of pDCs from baseline to 48 hours (0.41±0.18 to 0.36±0.18; p = 0.08), at 1 month (0.35±0.12; p = 0.06), and at 12-months (0.32±0.15; p = 0.06) (Table 2). This finding is of biological significance despite the small sample size.

**Table 2:** Proportions of circulating subsets of DCs (mDCs and pDCs) and their expression of adhesion molecules (VLA-4 and LFA-1) at baseline and during natalizumab therapy (NTZ = 6) in MS patients, healthy controls (HC = 10), and untreated RRMS controls (MSC = 4).

	HC	MSC	Baseline NTZ	48 h NTZ	1-mo NTZ	3-mo NTZ	12-mo NTZ
**MDCs**	0.27±0.22	1.03±1.03	0.51±0.50¥	0.55±0.54	0.61±0.50	0.20±0.20	0.45±0.32
**(HLA-DR^+^ BDCA-1)**	0.22 (0.82-0.49)	0.62 (0.35–2.12)	0.40 (0.10–0.89)	0.43 (0.17–0.90)	0.53 (0.28–0.93)	0.15 (0.17–0.42)	0.40 (0.13–0.82)
**VLA-4^+^ mDCs**	97.43±4.19	96.82±6.36	98.39±3.25	26.22±24.19*	23.80±28.32	8.58±13.99***	14.57±29.81***
	99.55 (95.39–100)	100 (90.46–100)	99.78 (97.21–100)	24.45 (1.20–49.69)	13.37 (0.57–54.18)	3.26 (0–15.71)	1.12 (0.63–25.41)
**LFA-1^+^ mDCs**	26.77±21.39	36.49±28.69	17.55±16.41	31.53±38.05	37.28±39.36	23.02±21.05	47.47±36.71*
	27.74 (4.53–45.22)	26.82 (15.72–66.92)	13.19 (4.21–30.83)	22.44 (0.49–60.82)	28.84 (3.17–73.11)	22.5 (2.81–40.86)	65.70 (0.80–74.74)
**pDCs**	0.53±0.29	0.53±0.06	0.41±0.18	0.36±0.18	0.35±0.12	0.52±0.50	0.32±0.15
**(CD123^+^HLA-DR^+^BDCA-2^+^)**	0.39 (0.32–0.87)	0.53 (0.46–0.59)	0.37 (0.25–0.56)	0.34 (0.22–0.49)	0.32 (0.25–0.49)	0.41 (0.23–0.75)	0.29 (0.18–0.51)
**VLA-4^+^ pDCs**	99.40±0.74	99.31±0.62*	97.00±6.22	13.41±31.35*	1.42±2.27	13.73±30.44***	0.28±0.51***
	99.54 (99.30–99.86)	99.38 (98.69–99.86)	99.37 (95.28–100)	0.82 (0.34–20.08)	0.52 (0.19–2.46)	1.47 (0.27–21.3)	0.00 (0.00–0.64)
**LFA-1^+^ pDCs**	9.64±8.23	1.95±1.16	2.27±1.62	4.88±9.43	2.83±3.59	4.37±7.71	3.05±3.08
	9.36 (1.21–18.49)	1.73 (1.00–3.1)	1.75 (0.87–3.95)	1.26 (0.69–7.32)	1.4 (0.28–5.48)	1.58 (0.43–6.89)	1.92 (1.37–5.3)

Data are presented as mean± standard deviation (SD), median (IQR). *Sig. (2-tailed) with respect to baseline by Wilcoxon Signed Ranks Test. ¥Sig. (2-tailed) with respect to healthy controls by Mann-Whitney U Test. *p<0.05; **p<0.01; ***p<0.001

**Figure 2:**
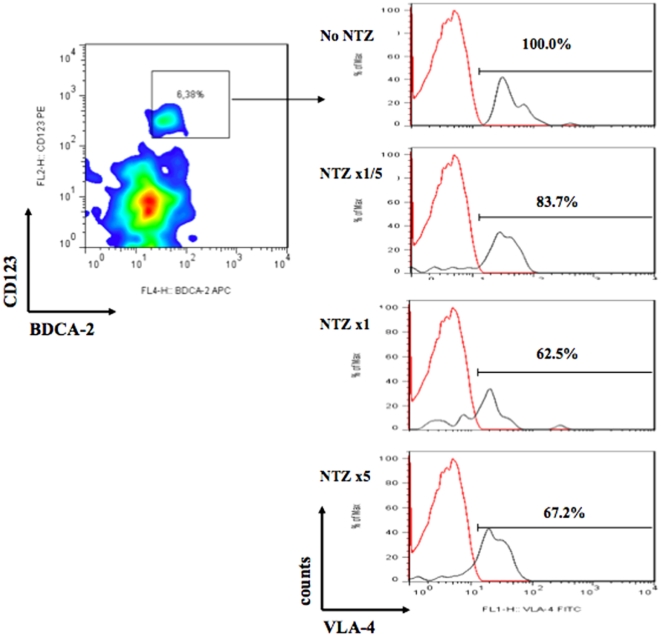
Dose-dependent down-modulation of VLA-4 expression on pDCs in vitro after 6 days of culture of PBMCs from healthy controls with 3 different doses of natalizumab (NTZ)—22 (×1/5), 110 (1×), and 550 µg/mL (5×), respectively—and antigen-specific stimulus (PPD, 50 IU/mL). The case shown is representative of 3 independent experiments. Isotypic IgG4 control is marked in red. These results were not reproduced in the MS patients tested (n = 3).

**Figure 3:**
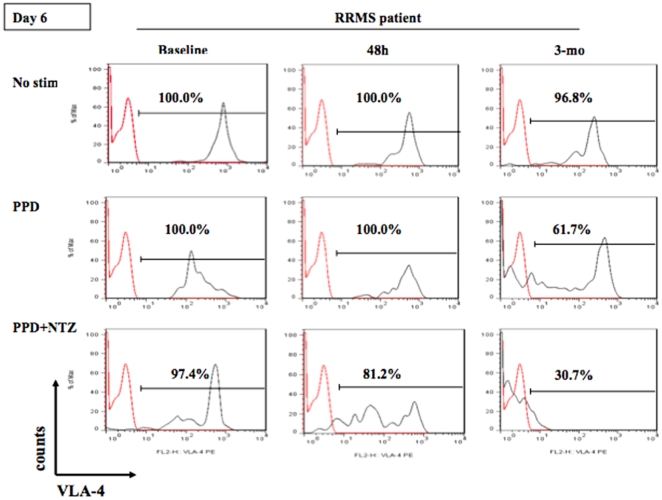
*In vitro* effect of natalizumab (NTZ) on VLA-4 expression on pDCs after 6 days of culture of PBMCs in the presence of antigen-specific stimulus (PPD) in NTZ-treated MS patients at different time-points (baseline, at 48 hours, and at 3 months). In vitro, VLA-4 expression on the surface of pDCs was greatly enhanced at day 6 with/without PPD, while NTZ was able to down-modulate it in NTZ-treated MS patients. *In vitro*, we observed that the enhanced expression of VLA-4 on pDCs was diminished in NTZ-treated MS patients more markedly after 3 months of therapy and that their pDCs were more sensitive *in vitro* to the VLA-4 down-modulating effects of NTZ after therapy. Isotypic IgG4 control is marked in red.

We cannot completely rule out the possibility that any lack of VLA-4 signal could be partly due to the effect of the drug covering or hiding the antigen and therefore rendering it not completely detectable by antibody staining. However, *in vitro* culture confirmed that, even at high doses of NTZ, we can observe VLA-4 expression levels in HC (Fig. 2) and NTZ-MS patients (Fig. 3).

### In vitro decrease in surface expression of VLA-4 on pDCs from HC and in NTZ-treated MS patient

Based on previous studies, as pDCs are key cells in the pathophysiology of MS and the subset that changed most specifically during NTZ treatment, we focused our studies on DCs.

We incubated pDCs from HC and NTZ-treated MS patients at baseline with increasing amounts of NTZ. We then performed *in vitro* cocultures of PPD-stimulated PBMCs with 3 increasing doses of NTZ (22 [1/5], 110 [1], and 550 [×5] µg/mL, respectively) and antigen-specific stimulus (PPD, 50 IU/mL) in HC and NTZ-treated MS. We observed that, in the presence of NTZ, pDCs displayed down-regulation of VLA-4 (83.7% to 62.5% and to 67.2%), respectively (mean of 3 independent analyses) in HC in a dose-dependent manner (day 6) (Fig. 2) (n = 3). However, in MS patients at baseline, *in vitro* VLA-4 expression on pDCs decreased uniformly (35% and 59%) in the 3 MS patients tested after administration of low NTZ doses (22 µg/mL n = 3), while higher NTZ doses were not able to further down-modulate VLA-4 expression with respect to PPD-stimulated PBMCs alone, suggesting a saturation effect (data not shown).

*In vitro* VLA-4 expression on unstimulated and PPD-stimulated pDCs in MS patients was highly up-modulated to 99% in NTZ-treated MS patients. Again, NTZ at low doses (22 µg/mL) in PPD-stimulated cultures was able to down-modulate VLA-4 expression on *ex vivo* pDCs from NTZ-treated MS patients at baseline, after 48 hours, and after 3 months of therapy (Figure 3). As shown in Figure 3 (in a representative experiment of 4 MS patients), the ability to up-modulate VLA-4 expression at day 6 in cultures of pDCs of MS patients treated for 3 months with NTZ was diminished from 61.7% to 30.7% at 3 months of therapy. In samples stimulated with PPD and to which NTZ was added at 48 hours and 3 months of therapy, VLA-4 expression decreased at a mean of 17% and 67% in 3 patients, respectively (from 97.9% to 81.2% at 48 h and to 32.3% at 3-months, respectively).

### NTZ decreases in vitro the functional ability of pDCs to stimulate CD4+ T-lymphocyte responses and antigen-specific proliferative responses

**Figure 4:**
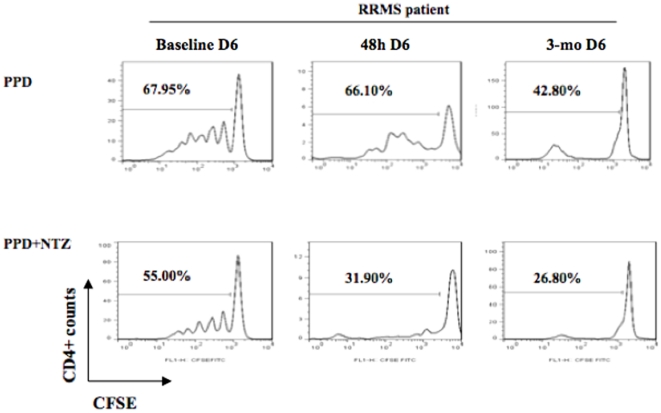
Effect of natalizumab (NTZ) on the immunogenic function of pDCs. CFSE-labelled autologous T lymphocytes from the same patient before NTZ treatment (baseline), at 48 hours, and at 3 months of NTZ therapy were cocultured with PPD-treated pDCs at a pDC∶PBMC ratio of 1∶10. After 6 days, lymphocytes were harvested, and the percentages of proliferating T cells (CFSElow) were calculated. In vitro, NTZ induced reduced CD4+ T-lymphocyte proliferation in PPD-specific responses in 4 individual NTZ-MS patients. The proliferative responses were weaker when we used pDCs from the same patients after 48 hours and 3 months of treatment with respect to baseline, suggesting an ongoing progressive functional impairment.

We also analyzed the capacity of pDC to induce proliferation of autologous T lymphocytes at day 6 of culture (CFSE-labelled autologous PBMCs were cocultured with PPD-treated pDCs at a pDC∶PBMC ratio of 1∶10 and low doses of NTZ (22 µg/mL) in NTZ-treated MS patients at baseline, 48 hours, and 3 months of NTZ. As shown in a representative experiment involving 4 patients (Figure 4), NTZ-treated pDCs were less able to stimulate PPD-specific CD4+ T cell proliferation than non–NTZ-treated pDCs at the 3 time-points, which ranged from ∼54%–76% inhibition of the PPD response in 3 different experiments in NTZ-treated MS patients. Moreover, VLA4 expression on pDcs is maximal without stimulus or with IVIG, while it is down-modulated after NTZ addition (data not shown). This effect was more marked in pDCs from the samples of the same patients monitored at 48 hours and 3 months of NTZ treatment (31.90% and 26.80%) (Figure 4). These results suggest that, in vivo, NTZ can modulate the ability of DC to induce antigen-specific CD4+ T-cell responses. Therefore, in addition to down-modulation of adhesion molecules and the subsequent reduced migration properties of pDCs, immunogenic function may be different. Our results differ from the study by Niino et al [14], in which *in vitro* addition of NTZ at very low doses (0.1 µg/mL), possibly negligible with respect to the serum NTZ trough concentrations in MS patients, seem to trigger a co-stimulatory signal, although increasing concentration resulted in lower enhancement of proliferation, compatible with our data.

## Discussion

We performed a prospective real-life observational 1-year study in a small series of RRMS patients treated with NTZ. Despite the small sample size, we show that NTZ induces a marked *in vivo* and *in vitro* decrease in surface expression of VLA-4 on pDCs and mDCs in the long term, a trend towards decreased pDCs proportions, and functional impairment of the interactions between T cells and pDCs.

Considering that DCs are the most potent APCs and play a critical role in promoting primary T- responses by bridging the gap between the pathogenesis of MS and CNS immunosurveillance, modifications after treatment with NTZ might provide important information about its beneficial effects on active MS. Immunohistochemical studies have demonstrated that resident and migrated DCs exist within the human CNS at the level of the meninges, choroid plexus, and vasculature [31], [32].

The most accepted hypothesis for the pathophysiology of MS is that CD4^+^ T cells are primed in the periphery and then enter the CNS. In the perivascular space, they encounter myelin antigen expressed by local APCs, such as microglia and DCs. Integrins on DCs, in particular VLA-4 and LFA-1, can potentially regulate endothelial extravasation, including trafficking across the BBB into the CNS, and differentiation, priming, and proliferation of immune cells both outside and inside the CNS. This finding may have implications for the pathogenesis of MS. Thus, local micromilieu signals at the level of the CNS determine functional differentiation in T cells driven by DCs, ranging from immunostimulatory responses to tolerogenic DC responses [33], [34], [35]. Importantly, we observed a decrease in the proportions of total pDCs (BDCA-2^+^HLA-DR^+^CD123^+^) at 12 months of therapy with NTZ.

The question of whether the therapeutic effects of NTZ in MS are mediated by inhibition of migration of DCs into the CNS or into other lymphoid tissues remains speculative. We hypothesize that migration of APCs, including DCs, and their expression of VLA-4 integrin molecules across the BBB into the CNS is critical for the initiation, local re-stimulation, and amplification of CD4^+^ and CD8^+^ T-cell-mediated immunity in the CNS [30]. CNS DCs may be key players in the control of local maintenance and amplification of T cell–mediated immunity. Indeed, NTZ might block the entry of activated DCs into the CNS by inhibiting the VLA-4 pathway—but not the LFA-1 pathway—in a similar way to other activated immune cells. Consequently, our data suggest a subsequent reduced presentation of self and foreign antigens by DCs to T in lymphoid tissues and locally within the CNS, where autoreactive lymphocytes were reactivated. Decreased entry of DCs and autoreactive immune cells into the CNS in RRMS patients receiving NTZ treatment may provide additional beneficial effects of NTZ leading to a decrease in MS activity [19], as revealed by our clinical and MRI findings. Conversely, under specific but as yet undefined circumstances, NTZ could facilitate the occurrence of PML or asymptomatic reactivation of JC virus [36]. According to this hypothesis, a recent study [37] of PML in an NZT-treated MS patient found lower numbers of DCs and CD4+ T cells in the cerebral perivascular spaces than in brain tissue from control patients with non-neurological diseases, patients with MS not treated with NTZ, and patients with PML not associated with NTZ therapy. The reason why some MS patients develop PML is unknown, but the immune competence of CD4+ and CD8+ T cells has been shown to be critical for control of JC virus infection. Thus, inefficient immune surveillance of the CNS by immune cells could favor the replication of latent JC virus in the CNS and, potentially, the clinical manifestation of its infection [38], [39]. In this scenario, a decrease in the presentation of antigens such as pDCs, which are capable of producing large amounts of type 1 interferons, could have an important role in the pathogenesis of conditions such as viral infection and autoimmune diseases (*eg,* MS). In MS, circulating pDCs show reduced production of IL-6 and IL-10 in response to *herpes simplex virus* type I, implying that their response to viral exposure could be impaired in MS [40]. Similarly, this mechanism could favor JC viral pathogenicity of the brain of NZT-treated MS patients with PML. Indeed, JC virus DNA has been found in oligodendrocytes and astrocytes in various regions of the normal brain. These findings suggest that JC virus has access to the brain in immunocompetent subjects. In the setting of marked immunosuppression, it is conceivable that resident virus may induce cytolysis in oligodendrocytes in some MS patients as a result of NTZ-impaired immune surveillance [41]). These effects on DCs are compounded by the effects of NTZ on CD4^+^ and CD8^+^ T cells, which also express the VLA-4 molecule.

In conclusion, although our cohort is small, this pilot study provides biologically significant original data that enable us to draw the following conclusions. First, in MS patients, circulating mDCs and pDCs express VLA-4 and LFA-1. Second, we observed a trend towards decreased proportions of circulating pDCs after treatment with NTZ, as well as decreased expression of VLA-4 on mDCs and pDCs. Third, functional impairment of the interaction between DCs and T cells during treatment with NTZ represents a potentially new therapeutic approach in MS. These data could point to a further mechanism of action for NTZ, and this may have significant implications for immune responses to self and non-self antigens both inside and outside the CNS and may thus provide additional clues for our understanding of the beneficial effects of NTZ. Further studies are needed to confirm our data and to ascertain whether NTZ also affects other functions of DCs. We suggest that monitoring clinical and MRI activity in patients with MS, together with analysis of immunological data, can help us to better understand the beneficial or potentially hazardous effects of NTZ in MS patients.
